# Association of Variants at 1q32 and *STAT3* with Ankylosing Spondylitis Suggests Genetic Overlap with Crohn's Disease

**DOI:** 10.1371/journal.pgen.1001195

**Published:** 2010-12-02

**Authors:** Patrick Danoy, Karena Pryce, Johanna Hadler, Linda A. Bradbury, Claire Farrar, Jennifer Pointon, Australo-Anglo-American Spondyloarthritis Consortium (TASC), Michael Ward, Michael Weisman, John D. Reveille, B. Paul Wordsworth, Millicent A. Stone, Spondyloarthritis Research Consortium of Canada (SPARCC), Walter P. Maksymowych, Proton Rahman, Dafna Gladman, Robert D. Inman, Matthew A. Brown

**Affiliations:** 1 The University of Queensland Diamantina Institute, Brisbane, Australia; 2 University of Oxford Institute of Musculoskeletal Sciences and NIHR Biomedical Research Centre, Nuffield Orthopaedic Centre, Oxford, United Kingdom; 3 National Institute of Arthritis and Musculoskeletal and Skin Diseases, National Institutes of Health, Bethesda, Maryland, United States of America; 4 Department of Medicine/Rheumatology, Cedars-Sinai Medical Centre, Los Angeles, California, United States of America; 5 Rheumatology and Clinical Immunogenetics, University of Texas Health Science Center at Houston, Houston, Texas, United States of America; 6 University of Bath, Bath, United Kingdom; 7 The University of Alberta, Edmonton, Alberta, Canada; 8 Memorial University, St. Johns, Newfoundland, Canada; 9 University of Toronto, Toronto, Ontario, Canada; Georgia Institute of Technology, United States of America

## Abstract

Ankylosing spondylitis (AS) is a common inflammatory arthritic condition. Overt inflammatory bowel disease (IBD) occurs in about 10% of AS patients, and in addition 70% of AS cases may have subclinical terminal ileitis. Spondyloarthritis is also common in IBD patients. We therefore tested Crohn's disease susceptibility genes for association with AS, aiming to identify pleiotropic genetic associations with both diseases. Genotyping was carried out using Sequenom and Applied Biosystems TaqMan and OpenArray technologies on 53 markers selected from 30 Crohn's disease associated genomic regions. We tested genotypes in a population of unrelated individual cases (n = 2,773) and controls (n = 2,215) of white European ancestry for association with AS. Statistical analysis was carried out using a Cochran-Armitage test for trend in PLINK. Strong association was detected at chr1q32 near *KIF21B* (rs11584383, *P* = 1.6×10^−10^, odds ratio (OR) = 0.74, 95% CI:0.68–0.82). Association with disease was also detected for 2 variants within *STAT3* (rs6503695, *P* = 4.6×10^−4^. OR = 0.86 (95% CI:0.79–0.93); rs744166, *P* = 2.6×10^−5^, OR = 0.84 (95% CI:0.77–0.91)). Association was confirmed for *IL23R* (rs11465804, *P* = 1.2×10^−5^, OR = 0.65 (95% CI:0.54–0.79)), and further associations were detected for *IL12B* (rs10045431, *P* = 5.2×10^−5^, OR = 0.83 (95% CI:0.76–0.91)), *CDKAL1* (rs6908425, *P* = 1.1×10^−4^, OR = 0.82 (95% CI:0.74–0.91)), *LRRK2/MUC19* (rs11175593, *P* = 9.9×10^−5^, OR = 1.92 (95% CI: 1.38–2.67)), and chr13q14 (rs3764147, *P* = 5.9×10^−4^, OR = 1.19 (95% CI: 1.08–1.31)). Excluding cases with clinical IBD did not significantly affect these findings. This study identifies chr1q32 and *STAT3* as ankylosing spondylitis susceptibility loci. It also further confirms association for *IL23R* and detects suggestive association with another 4 loci. STAT3 is a key signaling molecule within the Th17 lymphocyte differentiation pathway and further enhances the case for a major role of this T-lymphocyte subset in ankylosing spondylitis. Finally these findings suggest common aetiopathogenic pathways for AS and Crohn's disease and further highlight the involvement of common risk variants across multiple diseases.

## Author Summary

Ankylosing spondylitis is a common inflammatory arthritis primarily affecting the spine and pelvis. The disease is highly heritable (heritability>90%), and the major genetic allele for the disease, HLA-B27, contributes approximately half the genetic risk for the condition. Ankylosing spondylitis and inflammatory bowel disease (Crohn's disease and ulcerative colitis) frequently occur together in the same families and individuals, suggesting that they share common risk factors. We tested whether genes associated with Crohn's disease are also associated with ankylosing spondylitis and confirmed that the two diseases share associations at chromosome 1q32 near *KIF21B*, *STAT3*, *IL12B*, *CDKAL1*, *LRRK2/MUC19*, and chromosome 13q14. These associations were present even in ankylosing spondylitis cases with no clinical inflammatory bowel disease. These findings greatly expand our understanding of why these conditions co-occur and provide further evidence of pleiotropy in human disease pathology. As the genes *IL23R*, *STAT3*, and *IL12B* all influence Th17 lymphocyte differentiation/activation, this provides further evidence implicating the Th17 lymphocyte subset in the pathogenesis of ankylosing spondylitis.

## Introduction

Ankylosing spondylitis (AS) is one of a group of common inflammatory rheumatic diseases known as spondyloarthritidies in which involvement of the spine and sacroiliac joints is prominent [1]. Heritability of the disease assessed by twin studies has been determined to be >90% [2]. Apart from the well known *HLA-B27* association, recent genetic studies have identified *ERAP1*, *IL23R* and 2 intergenic regions at chr2p15 and chr21q22 as genes/loci associated with AS [3], [4]. However, these alleles only explain a fraction of the overall genetic risk for AS, and other loci are also expected to contribute to susceptibility.

There is increasing interest in the genetics community in the study of genetic findings from related diseases to identify pleiotropic genes, as an efficient method to identify further disease-associated variants. Findings from genome-wide association (GWA) studies have identified susceptibility genes common to different diseases, particularly in autoimmune conditions [5]. For example, variants in *PTPN22* are associated with rheumatoid arthritis (RA), type-1-diabetes (T1D) and Crohn's disease (CD). Thus far, only the gene *IL23R* (associated with AS) has also been found to be associated with inflammatory bowel disease (IBD) and psoriasis, although the three conditions commonly occur in the same patients, and are co-familial. About 10% of AS patients have overt IBD, and in addition about 70% of AS cases have subclinical terminal ileitis [6]. Gut inflammation is frequent in patients with spondylarthritis, and one-quarter of patients who have chronic spondyloarthritis have some features of CD [7]. Spondyloarthritis is also common in IBD patients. Axial and peripheral arthritis can occur in up to 30% of patients with IBD [8]. The prevalence of axial involvement in IBD is 10–20% for sacroiliitis and 3–12% for spondylitis [9], while radiographic evidence of sacroiliitis is reported in 10–51% of patients with IBD [10]. A study of families of AS probands (n = 205) and of healthy controls (n = 1,352) in the Icelandic population demonstrated evidence to support a common genetic component for AS and IBD [11]. In addition to confirming the known familiality of both conditions, the study demonstrated a risk ratio of 3.0 and 2.1 in 1^st^ and 2^nd^-degree relatives respectively, for the occurrence of AS in families of probands with IBD, and with the occurrence of IBD in families of patients with AS. It therefore seems likely that common pathogenic pathways may act in the development of both diseases and may be major players in chronic inflammatory disorders. We therefore sought to investigate CD risk variants for association with AS in order to explain the co-occurrence of both conditions.

## Results

This study has 80% power to detect an additive association (*P* = 0.05) with odds ratios of 1.15–1.23 for markers with minor allele frequencies of 0.5–0.1 respectively. These values are based on a disease prevalence of 0.4% in the general population and linkage disequilibrium between markers of r^2^ = 0.8.

In phase 1 of the genotyping experiment MAFs for 53 markers genotyped on cases were compared to MAF calculated from historical controls from the 1958 BBC (genotyped and imputed SNPs). Eight SNPs were excluded from further analysis; two variants failed to meet imputation QC thresholds, four were excluded based on their MAF and two failed the call rate criterion. Of the remaining markers, 23 SNPs achieved a nominal P value of 0.1 (Table S1). This is significantly more markers than would be expected by chance (*P* = 4.8×10^−10^). Of these, one failed assay design and 22 were taken forward for further genotyping in phase 2 of the experiment. A further marker failed QC thresholds in phase 2 and was excluded from further analysis.

Considering the combined phase 1 and 2 data, experiment-wise association (*P*<9.4×10^−4^) was observed with seven genomic regions (eight variants) and nominal association (*P*<0.05) was detected for 18 markers (Table 1). Associations were tested in both the overall cohort, and comparing cases with no clinical IBD (n = 2613) with healthy controls. The frequency of AS cases with coexistent IBD in our dataset is 5.8%. No significant difference was noted in any of the findings having excluded cases with clinical IBD, supporting these associations being relevant to the pathogenesis of AS, rather than just coexistent IBD.

**Table 1:** Association analysis findings of SNPs achieving P<0.1 in discovery cohort and successfully genotyped in the replication cohort.

Marker	Chr	Position	Candidate	Minor allele	Phase 1 Case MAF	Phase 1 Control MAF	Phase 1 P value	Phase 2 Case MAF	Phase 2 Control MAF	Phase 2 P value	Combined odds ratio(95% CI)	Combined P value	Combined excluding IBD P value
rs11465804	1	67475114	*IL23R*	G	0.042	0.061	4.4×10^−3^	0.035	0.053	3.9×10^−3^	0.65 (0.54–0.79)	1.2×10^−5^	5.8×10^−6^
rs6679677	1	114105331	*PTPN22*	A	0.074	0.092	3.1×10^−2^	0.095	0.106	0.23	0.88 (0.76–1.01)	6.4×10^−2^	0.14
rs11584383	1	199202489	Intergenic	C	0.26	0.319	7.9×10^−6^	0.242	0.296	6.7×10^−5^	0.74 (0.68–0.82)	1.6×10^−10^	1.0×10^−10^
rs12994997	2	233838242	*ATG16L1*	G	0.456	0.493	1.1×10^−2^	0.471	0.476	0.72	0.91 (0.84–0.99)	2.4×10^−2^	4.2×10^−2^
rs2241880	2	233848107	*ATG16L1*	T	0.453	0.491	8.8×10^−3^	0.465	0.477	0.43	0.9 (0.83–0.98)	1.0×10^−2^	2.1×10^−2^
rs3792106	2	233855479	*ATG16L1*	A	0.395	0.435	5.7×10^−3^	0.406	0.413	0.66	0.9 (0.83–0.98)	1.1×10^−2^	1.5×10^−2^
rs3924462	3	49499240	*MST1*	C	0.406	0.442	1.2×10^−2^	0.435	0.453	0.26	0.91 (0.84–0.99)	5.0×10^−2^	7.8×10^−2^
rs3181219	5	158684717	*IL12B*	T	0.1	0.118	5.0×10^−2^	0.109	0.116	0.43	0.88 (0.78–1)	5.3×10^−2^	5.5×10^−2^
rs1433048	5	158688423	*IL12B*	G	0.165	0.191	2.2×10^−2^	0.172	0.176	0.72	0.9 (0.81–1)	5.0×10^−2^	3.9×10^−2^
rs10045431	5	158747111	*IL12B*	A	0.257	0.302	6.7×10^−4^	0.259	0.287	3.7×10^−2^	0.83 (0.76–0.91)	5.2×10^−5^	1.1×10^−4^
rs6908425	6	20836710	*CDKAL1*	T	0.194	0.229	4.0×10^−3^	0.19	0.216	3.2×10^−2^	0.82 (0.74–0.91)	1.1×10^−4^	1.0×10^−4^
rs2301436	6	167357978	*CCR6*	G	0.507	0.469	8.0×10^−3^	0.448	0.461	0.39	1.03 (0.95–1.12)	0.44	0.44
rs1456893	8	50240218	Intergenic	G	0.291	0.318	5.2×10^−2^	0.306	0.334	4.5×10^−2^	0.89 (0.82–0.97)	1.1×10^−2^	1.2×10^−2^
rs10758669	9	4971602	*JAK2*	C	0.353	0.327	6.5×10^−2^	0.37	0.343	7.5×10^−2^	1.14 (1.05–1.24)	2.9×10^−4^	2.3×10^−3^
rs11190140	10	101281583	*NKX2-3*	C	0.518	0.482	1.3×10^−2^	0.494	0.509	0.34	1.11 (1.02–1.2)	1.3×10^−2^	3.5×10^−2^
rs11175593	12	38888207	*LRRK2/MUC19*	T	0.02	0.013	5.3×10^−2^	0.025	0.01	5.8×10^−4^	1.92 (1.38–2.67)	9.9×10^−5^	1.0×10^−4^
rs3764147	13	43355925	Intergenic	G	0.235	0.202	4.3×10^−3^	0.237	0.214	5.9×10^−2^	1.19 (1-08–1.31)	5.9×10^−4^	9.1×10^−4^
rs2306580	17	37745206	*STAT3*	G	0.065	0.085	9.8×10^−3^	0.07	0.076	0.43	0.83 (0.71–0.96)	2.3×10^−2^	2.6×10^−2^
rs6503695	17	37753059	*STAT3*	C	0.329	0.352	9.7×10^−2^	0.32	0.37	6.9×10^−4^	0.86 (0.79–0.93)	4.6×10^−4^	3.4×10^−4^
rs4103200	17	37760591	*STAT3*	C	0.258	0.284	4.5×10^−2^	0.254	0.284	2.4×10^−2^	0.86 (0.79–0.95)	1.9×10^−3^	2.0×10^−3^
rs744166	17	37767727	STAT3	C	0.399	0.437	7.3×10^−3^	0.395	0.446	7.9×10^−4^	0.84 (0.77–0.91)	2.6×10^−5^	3.8×10^−5^

Positions based on NCBI reference sequence build 36.3. MAF, minor allele frequency; chr, chromosome. Phase 1 involved 1230 cases and 1295 controls, and phase 2 1545 cases and 920 controls.

The strongest association was identified at genome-wide significance for rs11584383 at chr1q32 (*P* = 1.6×10^−10^) with OR = 0.74 (95% CI:0.68–0.82). This marker is downstream of and flanked by *KIF21B* and the putative open reading frame *C1orf106*. Convincing evidence of association was also identified for *STAT3*. Association in *STAT3* was detected at experiment-wise significance for 2 markers (rs744166, *P* = 2.6×10^−5^, OR = 0.84 (95% CI:0.77–0.91); rs6503695, *P* = 4.6×10^−4^, OR = 0.86 (95% CI:0.79–0.93)). Another 2 variants within the gene showed more modest association (rs2306580, *P* = 0.023; rs4103200, *P* = 1.9×10^−3^) but rs4103200 demonstrated nominal association in both phases of the genotyping experiment.

Variants within or near *CDKAL1* (rs6908425, *P* = 1.1×10^−4^, OR = 0.82 (95% CI:0.74–0.91)), *IL12B* (rs10045431, *P* = 5.2×10^−5^, OR = 0.83 (95% CI:0.76–0.91)), *LRRK2/MUC19* (rs11175593, *P* = 9.9×10^−5^, OR = 1.92 (95% CI:1.38–2.67)) and at chr13q14 within *C13orf31* (rs3764147, *P* = 5.9×10^−4^, OR = 1.19 (95% CI:1-08–1.31)) also demonstrated experiment-wise significant association in this study. The function of CDKAL1 is poorly understood but the gene has been associated with type-2-diabetes [12], [13], and could also possibly be a psoriasis risk locus [14].

Other nominal associations detected in this study include 3 variants within *ATG16L1* and single SNPs at *MST1*, *JAK2*, *NKX2-3* and chr8q24. Further studies will be required to robustly establish the significance of these findings. No association was observed at *TNFSF15* (rs4263839, *P* = 0.3), consistent with previous findings in AS [3], [4], contrasting with findings in undifferentiated spondyloarthritis [15], in which suggestive but not genome-wide significant association has been reported (rs4979459, *P* = 4.9×10^−5^). The peak IBD and spondyloarthritis associations are with different SNPs that lie 85kb apart, and thus if this gene is truly spondyloarthritis-associated, it is likely that different genetic variants are associated with IBD and spondyloarthritis.

This study also confirms the *IL23R* association with AS at rs11465804 (*P* = 1.2×10^−5^), OR = 0.65 (95% CI:0.54–0.79). In a recent study, *IL23R* association with AS was confirmed with a peak signal within the gene at rs11209026 (*P* = 9.1×10^−14^) [4]. However, evidence of association at rs11465804 (3,432 bp away from rs11209026) is much weaker, and was previously reported at *P* = 2×10^−4^ in a study involving 1471 AS cases and 2125 matched control individuals [3].

## Discussion

The study presented here has identified new loci associated with AS. The strongest of these associations was within an intergenic region at chr1q32, near the gene *KIF21B*. The protein encoded by this gene belongs to a family of kinesin motor proteins. Kinesins are used for the transport of essential components along axonal and dendritic microtubules by neurons. *KIF5A* has been associated with rheumatoid arthritis, type-1-diabetes, and is close to a locus recently reported to be associated with multiple sclerosis [16]. It is possible that *KIF5A* is not the key associated gene at this chromosome 12q13-14 locus. However, if confirmed as the true disease-susceptibility gene for these autoimmune diseases, this would strongly suggest alternate functions for the kinesin protein family.

The *STAT3* association is particularly significant because of its role, along with IL23R, in the Th17 pathway. In response to cytokine signaling through the IL-23R, STAT3 is activated by phosphorylation and is translocated to the nucleus where it acts as a transcriptional activator. Loss of function mutations of *STAT3* result in Job's syndrome, in which an absence of Th17 lymphocytes leads to recurrent severe infections, particularly with extracellular bacteria [17].

The association with *IL12B* is of particular interest given the associations of *IL23R* and *STAT3* with AS. This gene has also been shown to be associated with psoriasis in Caucasian and Chinese populations [18], [19]. *IL12B* encodes the p40 subunit common to both IL-12 and IL-23 and again highlights the involvement of Th17 cells in disease development. Another marker (rs1433048) within the gene was also nominally associated (*P*<0.05) in the combined analysis of the study. It is not clear at the *LRRK2/MUC19* locus which is the key associated gene with Crohn's disease, although a recent study suggests that *LRRK2* is the more likely to be truly disease-associated [20]. LRRK2, a member of the leucine-rich repeat kinase family, is thought to be involved in the process of autophagy. *MUC19* encodes a mucin involved in epithelial lining protection; altered intestinal permeability has long been thought to be important in the pathogenesis of AS.

This study also provides further evidence of pleiotropic effects in human disease pathology. A notable example of this is the association of *PTPN22* with several autoimmune conditions including RA, T1D, CD and SLE. One of the 1^st^ AS risk loci identified, *IL23R*, is also associated with both forms of IBD (ulcerative colitis and Crohn's disease) as well as psoriasis. In this study we provide further evidence for previously and newly identified pleiotropic genes in autoimmune diseases. Given the delicate nature of the immune system and the tight control of the different cell populations it is not surprising that risk alleles of important immune response genes may be associated across a number of different conditions. These findings support the use of study designs focusing on genes previously identified as being associated with related conditions as being an efficient method for identifying further genetic disease-associations.

This study of genes associated with Crohn's disease has identified definite genome-wide significant association with AS of SNPs at chromosome 1q32 near *KIF21B*, and experiment-wise association at five other novel-AS loci including *STAT3*, *IL12B*, *CDKAL1*, *LRRK2/MUC19*, and at chr13q14. This confirms that genes play an important part in the co-familiality of Crohn's disease and AS, and highlights the value of studies of potentially pleiotropic genes in related diseases.

## Materials and Methods

Cases included in the study were unrelated individuals with AS of white European ancestry from the UK, USA, Canada and Australia. The diagnosis of AS met the modified New York definition criteria [21]. Ethnically matched unrelated control individuals were selected from the 1958 British Birth Cohort (BBC) and an Oxfordshire (UK) healthy blood donor cohort. All patients gave informed, written consent, and the study was approved by the relevant ethics review committees.

SNPs chosen for the study were selected from previously reported risk loci identified in a CD GWA study [22]. Three candidate loci of high interest were studied in greater depth (*STAT3*, *IL12B* and *ATG16L1*). For these genes, tagging SNPs (r^2^≥0.8) were selected using Tagger in Haploview (http://www.broadinstitute.org/haploview/haploview) and using the CEU population as a reference panel from the International HapMap Project database (http://www.hapmap.org/). Whenever assay design for selected SNPs failed, perfect proxy SNPs (r^2^ = 1) were selected.

Genotyping was carried out in two phases. In phase 1, 53 SNPs from 30 distinct genomic regions were selected for genotyping in 1,230 cases. Case genotype results were compared with historical genotypes from 1,295 unrelated individuals from the 1958 British Birth Cohort, which had been typed with both the Illumina HumanHap 550 and Affymetrix SNP Array 5.0 microarrays. Whenever selected markers were missing from the control genotypes, imputation was carried out using MACH (http://www.sph.umich.edu/csg/abecasis/MACH/index.html) against the HapMap CEU dataset (reference panel). Imputed markers with low overall quality scores (Q<0.95) and/or in low LD with typed markers (r^2^<0.3) were excluded from further analysis. Only genotypes with quality scores ≥0.95 were included in the study. All nominally associated markers (*P*<0.1) were taken forward for genotyping in phase 2 on 1,543 cases and 920 controls.

In phase 2, genotyping was performed using Sequenom (Sequenom Inc., San Diego, USA), and Applied Biosystems TaqMan and OpenArray technologies (Life Technologies, Carlsbad, USA). For Sequenom, SNPs were assayed and typed using iPLEX chemistry on a matrix assisted laser desorption/ionization time-of-flight (MALDI_TOF) mass spectrometer. PCR reactions, cycling conditions and post-PCR extension reactions were all performed as recommended by the manufacturer. The iPLEX reaction products were desalted and spotted on SpectroChip and were processed and analysed in a compact mass spectrometer (MassARRAY Workstation). MassARRAY Typer 4.0 software was used for automated and manual genotype calling. OpenArray is a new genotyping platform technology from Applied Biosystems for medium-throughput experiment. SNPs were typed using TaqMan genotyping chemistry supported on a metal-based array. DNA samples were loaded and amplified on arrays as recommended by the manufacturer. Arrays were scanned on the OpenArray NT imager and genotypes were called using the OpenArray SNP Genotyping analysis software. Whenever assay design or the genotyping assay failed, markers were then genotyped using single TaqMan probe technology as recommended by the manufacturer.

Association statistics were calculated using PLINK software [23]. Analysis was carried out using the Cochran-Armitage test for trend, excluding markers failing the following criteria; missingness rate >0.1, minor allele frequency (MAF)<0.01 and exact Hardy-Weinberg equilibrium *P*<5×10^−5^. We also excluded individuals with a missing genotype rate >0.1 from the analysis. Experiment-wise significance (*P* = 9.4×10^−4^) was determined by Bonferroni correction based on the total number of markers genotyped in phase1 of the study.

## Supporting Information

Table S1 Association study findings for phase 1 for all markers genotyped. Markers with blank case and control MAF failed genotyping. Positions based on NCBI reference sequence build 36.3. MAF, minor allele frequency; chr, chromosome.
(0.09 MB DOC)
