# Supplementary material for: Association of Variants at 1q32 and STAT3 with Ankylosing Spondylitis Suggests Genetic Overlap with Crohn's Disease
Source: PLoS Genet. 2010 Dec 2;6(12):e1001195. doi: 10.1371/journal.pgen.1001195 (PMC2996314; doi:10.1371/journal.pgen.1001195)
Supplement: Table S1 — Association study findings for phase 1 for all markers genotyped. Markers with blank case and control MAF failed genotyping. Positions based on NCBI reference sequence build 36.3. MAF, minor allele frequency; chr, chromosome. (0.09 MB DOC) [file pgen.1001195.s001.doc]

Supplementary Table: Association study findings for phase 1 for all markers genotyped. Markers with blank case and control MAF failed genotyping.

| Marker | Chr | Position | Candidate gene | Minor allele | Major allele | Case MAF | Control MAF | χ2 | P value |
| --- | --- | --- | --- | --- | --- | --- | --- | --- | --- |
| rs11465804 | 1 | 67475114 | *IL23R* | G | T | 0.042 | 0.061 | 8.1 | 4.4x10^-3^ |
| rs6679677 | 1 | 114105331 | *PTPN22* | A | C | 0.074 | 0.092 | 4.7 | 3.1x10^-2^ |
| rs2274910 | 1 | 159118670 | *ITLN1* | T | C | 0.32 | 0.33 | 0.2 | 0.68 |
| rs9286879 | 1 | 171128857 |  | G | A | 0.24 | 0.25 | 0 | 0.96 |
| rs11584383 | 1 | 199202489 |  | C | T | 0.26 | 0.32 | 20 | 7.9x10^-6^ |
| rs1441090 | 2 | 233832781 | *ATG16L1* | A | G | 0.069 | 0.073 | 0.3 | 0.59 |
| rs6758317 | 2 | 233833690 | *ATG16L1* | T | C | 0.19 | 0.20 | 1.5 | 0.22 |
| rs12994997 | 2 | 233838242 | *ATG16L1* | G | A | 0.46 | 0.49 | 6.5 | 1.1x10^-2^ |
| rs12105443 | 2 | 233843767 | *ATG16L1* | T | C |  |  |  |  |
| rs2289473 | 2 | 233846764 | *ATG16L1* | A | G | 0.061 | 0.070 | 1.6 | 0.21 |
| rs2241880 | 2 | 233848107 | *ATG16L1* | T | C | 0.45 | 0.49 | 6.9 | 8.9x10^-3^ |
| rs7585217 | 2 | 233854145 | *ATG16L1* | A | G |  |  |  |  |
| rs3792106 | 2 | 233855479 | *ATG16L1* | A | G | 0.40 | 0.44 | 7.6 | 5.7x10^-3^ |
| rs7587051 | 2 | 233859494 | *ATG16L1* | G | C |  |  |  |  |
| rs4663136 | 2 | 233862456 | *ATG16L1* | C | G | 0.29 | 0.31 | 2 | 0.16 |
| rs6754677 | 2 | 233864200 | *ATG16L1* | G | A | 0.35 | 0.37 | 2.9 | 8.7x10^-2^ |
| rs11682236 | 2 | 233868479 | *ATG16L1* | G | A | 0.057 | 0.055 | 0.1 | 0.75 |
| rs3924462 | 3 | 49499240 | *MST1* | C | A | 0.41 | 0.44 | 6.3 | 1.2x10^-2^ |
| rs4613763 | 5 | 40428485 | *PTGER4* | C | T | 0.13 | 0.13 | 0.1 | 0.76 |
| rs2188962 | 5 | 131798704 |  | T | C | 0.43 | 0.44 | 0.3 | 0.58 |
| rs11747270 | 5 | 150239060 | *IRGM* | G | A | 0.084 | 0.072 | 2.6 | 0.11 |
| rs3213097 | 5 | 158681257 | *IL12B* | T | A | 0.21 | 0.19 | 3.3 | 6.8x10^-2^ |
| rs3213096 | 5 | 158682907 | *IL12B* | T | C |  |  |  |  |
| rs3181219 | 5 | 158684717 | *IL12B* | T | C | 0.10 | 0.12 | 3.9 | 5.0x10^-2^ |
| rs3181216 | 5 | 158685556 | *IL12B* | T | A | 0.33 | 0.31 | 0.9 | 0.338 |
| rs1433048 | 5 | 158688423 | *IL12B* | G | A | 0.17 | 0.19 | 5.3 | 2.2x10^-2^ |
| rs10045431 | 5 | 158747111 | *IL12B* | A | C | 0.26 | 0.30 | 11.6 | 6.7x10^-4^ |
| rs6908425 | 6 | 20836710 | *CDKAL1* | T | C | 0.19 | 0.23 | 8.3 | 4.0x10^-3^ |
| rs7746082 | 6 | 106541962 |  | C | G |  |  |  |  |
| rs2301436 | 6 | 167357978 | *CCR6* | G | A | 0.51 | 0.47 | 7 | 8.0x10^-3^ |
| rs1456893 | 8 | 50240218 |  | G | A | 0.29 | 0.32 | 3.8 | 5.2x10^-2^ |
| rs10758669 | 9 | 4971602 | *JAK2* | C | A | 0.35 | 0.33 | 3.4 | 6.5x10^-2^ |
| rs4263839 | 9 | 116606261 | *TNFSF15* | A | G | 0.32 | 0.33 | 0.8 | 0.38 |
| rs1551398 | 9 | 126609233 |  | C | T | 0.38 | 0.39 | 0.4 | 0.54 |
| rs17582416 | 10 | 35327656 |  | G | T | 0.35 | 0.34 | 0.9 | 0.35 |
| rs10995271 | 10 | 64108492 | *ZNF365* | C | G | 0.38 | 0.39 | 0.5 | 0.50 |
| rs11190140 | 10 | 101281583 | *NKX2-3* | C | T | 0.52 | 0.48 | 6.2 | 1.3x10^-2^ |
| rs7927894 | 11 | 75978964 |  | T | C | 0.41 | 0.40 | 0 | 0.830 |
| rs11175593 | 12 | 38888207 | *LRRK2/MUC19* | T | C | 0.020 | 0.013 | 3.8 | 5.3x10^-2^ |
| rs3764147 | 13 | 43355925 |  | G | A | 0.24 | 0.20 | 8.1 | 4.3x10^-3^ |
| rs2076756 | 16 | 49314382 | *NOD2/CARD15* | G | A | 0.26 | 0.25 | 0.5 | 0.49 |
| rs2872507 | 17 | 35294289 | *ORMDL3* | A | G | 0.47 | 0.49 | 1 | 0.33 |
| rs1053023 | 17 | 37719142 | *STAT3* | C | T | 0.19 | 0.20 | 1 | 0.33 |
| rs2293152 | 17 | 37735055 | *STAT3* | C | G |  |  |  |  |
| rs2306580 | 17 | 37745206 | *STAT3* | G | C | 0.065 | 0.085 | 6.7 | 9.8x10^-3^ |
| rs7215104 | 17 | 37745542 | *STAT3* | T | C |  |  |  |  |
| rs6503695 | 17 | 37753059 | *STAT3* | C | T | 0.33 | 0.35 | 2.8 | 9.6x10^-2^ |
| rs4103200 | 17 | 37760591 | *STAT3* | C | G | 0.26 | 0.28 | 4 | 4.5x10^-2^ |
| rs744166 | 17 | 37767727 | *STAT3* | C | T | 0.40 | 0.44 | 7.2 | 7.3x10^-3^ |
| rs6503698 | 17 | 37787690 | *STAT3* | T | C |  |  |  |  |
| rs2542151 | 18 | 12769947 | *PTPN2* | G | T | 0.18 | 0.16 | 2.7 | 0.10 |
| rs1736020 | 21 | 15734423 |  | A | C | 0.42 | 0.43 | 0.1 | 0.77 |
| rs743479 | 21 | 44436378 | *ICOSLG* | G | A | 0.42 | 0.40 | 1.5 | 0.22 |

Positions based on NCBI reference sequence build 36.3. MAF, minor allele frequency; chr, chromosome.
